# New Ti-Alloys and Surface Modifications to Improve the Mechanical Properties and the Biological Response to Orthopedic and Dental Implants: A Review

**DOI:** 10.1155/2016/2908570

**Published:** 2016-01-14

**Authors:** Yvoni Kirmanidou, Margarita Sidira, Maria-Eleni Drosou, Vincent Bennani, Athina Bakopoulou, Alexander Tsouknidas, Nikolaos Michailidis, Konstantinos Michalakis

**Affiliations:** ^1^Department of Prosthodontics, Aristotle University of Thessaloniki, Faculty of Health Sciences, School of Dentistry, 54124 Thessaloniki, Greece; ^2^Aristotle University of Thessaloniki, Faculty of Health Sciences, School of Medicine, 54124 Thessaloniki, Greece; ^3^Department of Prosthodontics, University of Otago, School of Dentistry, Dunedin 9054, New Zealand; ^4^Aristotle University of Thessaloniki, School of Engineering, Department of Mechanical Engineering, Physical Metallurgy Laboratory, 54124 Thessaloniki, Greece; ^5^Division of Graduate and Postgraduate Prosthodontics, Department of Prosthodontics, Tufts University School of Dental Medicine, Boston, MA 02111, USA

## Abstract

Titanium implants are widely used in the orthopedic and dentistry fields for many decades, for joint arthroplasties, spinal and maxillofacial reconstructions, and dental prostheses. However, despite the quite satisfactory survival rates failures still exist. New Ti-alloys and surface treatments have been developed, in an attempt to overcome those failures. This review provides information about new Ti-alloys that provide better mechanical properties to the implants, such as superelasticity, mechanical strength, and corrosion resistance. Furthermore, in vitro and in vivo studies, which investigate the biocompatibility and cytotoxicity of these new biomaterials, are introduced. In addition, data regarding the bioactivity of new surface treatments and surface topographies on Ti-implants is provided. The aim of this paper is to discuss the current trends, advantages, and disadvantages of new titanium-based biomaterials, fabricated to enhance the quality of life of many patients around the world.

## 1. Introduction

Titanium was discovered in 1791 by an amateur mineralogist named William Gregor, in magnetic iron-sand (ilmenite). This element was also identified in 1795 by the German chemist Martin Heinrich Klaproth, who named it “titanium” after the Titans in Greek mythology, the powerful sons of Earth. Pure titanium is one of the most abundant metals on Earth's crust and lithosphere, but it could not be isolated in large amount until the invention of the Kroll process by a metallurgist named William Kroll, in 1946 [1]. Post-World War II advances enabled applications of titanium in medical surgical and dental devices.

Nowadays, commercially pure titanium (cp-Ti) and its alloys are widely used for manufacturing orthopedic and dental implants due to their superior mechanical and physical properties, such as corrosion resistance and high modulus of elasticity in tension, and their excellent biocompatibility [2]. There are four grades of cp-Ti depending on their content to oxygen and iron [3]. Following cp-Ti, Ti-6Al-4V, which is also known as Ti6-4 and Ti-grade 5, became commonly used for biomedical applications (i.e., orthopedic and dental implants), because of its enhanced mechanical strength [4]. It is well known that one main reason for the excellent physical and biological properties of titanium and its alloys is the native oxide film (TiO_2_) that is created spontaneously on its surface upon air exposure [5]. This film, having only a few nanometers thickness (4.3 ± 0.2 nm for the mechanically polished cp-Ti surface) [6], appears to be responsible for the chemical stability, chemical inertness, corrosion resistance, and even biocompatibility of titanium [5].

Unquestionably, much progress has been done over the years, and the survival rates of dental and orthopedic implants are quite satisfactory. Specifically, for dental implants, survival rates range from 90% to 96.5% [7–10], whereas for orthopedic implants the same rates are reported as follows: 80–94% at 15 years for total hip arthroplasty (THA) [11], 98.4–98.7% at 10 years for total knee prosthesis (TKP) [12], 91% at 10 years for shoulder arthroplasty [13], and 53% and 90% at 5 years for total elbow arthroplasty (TEA) in patients with posttraumatic arthritis or fractures and inflammatory arthritis, respectively [14]. Reasons for failures in all of the above studies are infection, implant fractures, wear of the articulating surfaces, and implant loosening that can be attributed to stress-shielding effect, septic or aseptic inflammation, material fatigue, and excessive activity by the patient and debonding at the tissue-implant interface. Despite the satisfactory results, there is a need for improvement. For instance, between 2005 and 2030 total arthroplasty revision surgeries are estimated to increase at 137% and 607% for hip and knee revision surgeries, respectively [15].

Corrosion is a phenomenon closely related to implant failures [16]. The procedure of corrosion begins after its in vivo implantation of a material in the human body and its contact with the extracellular body fluids [17]. The human body is a hostile environment containing water, complex organic compounds, proteins, amino acids, lymph, saliva, plasma, and a variety of ions, such as sodium, chloride, bicarbonate, oxygen, potassium, calcium, magnesium, and chloride. Upon implantation, corrosion is induced by electrochemical interactions between the implant material and the mentioned chemical compounds [17]. As a result, pitting, fretting, galvanic corrosion, and stress corrosion occur, while the following complications are both mechanical and biological.

Mechanical complications include mainly fatigue fracture and they are accelerated by corrosion [16, 17]. More specifically, concerning dental implants the percentage of fractures of the material is reported between 0.2% and 1.5%, in follow-up intervals up to 15 years [18–20]. Similarly, fractures of metallic implant components were reported at 4.2% in a study that included 142 consecutive cases of cervical fusion, after an average follow-up interval of at least 3 years [21]. Furthermore 2.3% of 219 distally fixed femoral stems were fractured during a time interval from 1 to 6 years in a prospective study [22]. Lakstein et al. reported that one (1.4%) out of 69 patients with total hip arthroplasty needed revision after follow-up periods ranging from 5 to 9.5 years [23].

Biological complications related to corrosion are caused by the metal ion release and include toxicity, carcinogenicity, and hypersensitivity [17]. Biocorrosion, tribocorrosion, and their combination result in release of metallic particles from the implant material to peri-implant tissues and other body organs [24–27]. This phenomenon is more intense when biofilms or high concentrations of fluoride are present, a common situation in the oral environment [28]. Specifically, the presence of metallic ions activates macrophages, neutrophils, and T-lymphocytes and provokes enhanced output of cytokines and metallic proteases [29, 30]. Furthermore, in the case of Ti-6Al-4V, aluminum and vanadium particles have been characterized as toxic and mutagenic, respectively, and have been accused for osteomalacia, Alzheimer's disease, and neurological disorders [31–35].

The stress-shielding effect is another phenomenon related to implant failure [36]. In the case of orthopedic implants, the bone is not properly loaded, because of implant rigidity and, consequently, the implant is progressively loosened, due to bone atrophy [36, 37]. It seems that this situation is inhibited or at least decelerated with the use of more elastic implant alloys [38]. In particular, Young's modulus value for the bone ranges between 6.9 ± 4.3 GPa and 25.0 ± 4.3 GPa [39–41], while the same value for cp-Ti and Ti-6Al-4V is 103–107 GPa and 114–120 GPa, respectively [42]. It can be assumed that there is a need for more elastic Ti-alloys to be used as orthopedic implant materials.

As a bioinert material, titanium allows close apposition of bone under proper conditions. This procedure is called osseointegration [43] and begins with the absorption of ions, proteins, polysaccharides, and proteoglycans by the Ti-oxide layer [44, 45]. Afterwards, macrophages, neutrophils, and osteoprogenitor cells migrate on the bone-implant interface and lead to bone apposition in close contact with the implant surface [45]. Although a direct contact between bone and implant can be observed, this situation is not common. More often, there is a thin amorphous zone or lamina limitans [45–47], which appears to have a thickness of 20–50 nm [46], or according to other studies it is larger and does not exceed 400 nm [47]. The osseointegration procedure takes a time period of at least 3–5 months to be adequate [44], a fact that often complicates immediate loading of orthopedic and dental implants.

Consequently, there is a need for new Ti-alloys and surface treatments with the following characteristics:

- High corrosion resistance, lower modulus of elasticity, high mechanical strength, and wear resistance to avoid mechanical failures.
- Better biocompatibility, without allergic reactions, cytotoxicity, and carcinogenicity, in order to avoid biological failures.
- More bioactive surfaces that will lead to faster and more enhanced osseointegration.
- Increased antimicrobial properties that will reduce failures due to infection.

## 2. New Ti-Alloys

### 2.1. Porous Ti-Alloys

Porous surfaces seem to induce bone ingrowth [48, 49]. Based on that concept, Ti-implants with different porosities have been introduced [50–55]. Two types of pores can be detected by SEM observation on a porous Ti-implant surface: (a) macropores (>100 *μ*m) that are created by the use of space holders and (b) small micropores (~10 *μ*m) that can be observed on the walls of the macropores and arise during the sintering process [51, 55]. However, the optimal pore size facilitating cell colonization still remains unanswered and findings from different studies are ambiguous and conflicting. Xue et al. conducted MTT assays of porous cp-Ti samples (27% porosity, pores size between 100 and 800 *μ*m) versus nonporous ones on cloned osteoblastic precursor cell lines 1 (OPC1), derived from human fetal bone tissue [51]. Cell numbers were significantly higher on porous Ti-surfaces after 3 days (*p* < 0.01) and 10 days (*p* < 0.05), whereas after 21 days porous surfaces had still more cells but there was no statistically significant difference compared to the nonporous Ti-sample. The same authors demonstrated that cells could not grow into pores that are sized less than 100 *μ*m, whereas cell bridges were formed between elongations of adjacent cells in pores that are sized 150 *μ*m. In pores larger than 200 *μ*m the cells grew into the center of the pores without forming cell bridges. Furthermore, Hollander et al. tested in vitro the performance of porous Ti-6Al-4V samples fabricated by direct laser forming (DLF) on human primary osteoblast cultures (HOB) [54]. After 14 days, live/dead staining showed complete overgrowth of cells in pores that are sized 500 *μ*m. On the contrary, pores with sizes 700 *μ*m and 1000 *μ*m were not completely overgrown but led to circular growth patterns.

Interconnectivity between pores has also been tested as a factor affecting bone ingrowth into porous implants [52]. Otsuki et al. evaluated 3D bone ingrowth on four sintered porous Ti-samples using micro-CT [52]. Ammonium hydrogen was used as a space holder. The samples (50% porosity, 250–500 *μ*m pores size) were implanted into femoral condyles of male rabbits for 6 weeks. The authors concluded that pore throats narrower than 52 *μ*m did not allow bone ingrowth. However, they highlighted that different Ti-alloys with different surface treatments may demonstrate different threshold values.

Attempts have been made to describe the relationship between the density and structure of porous materials and their mechanical properties [56–58]. A simplified model to express the relationship between porosity and mechanical properties has been developed [59]:

(1) E∗Es=C1ρ∗ρsm1,σ∗σs=C2ρ∗ρsm2,

where *E*, *σ*, and *ρ* correspond to elastic modulus, strength, and density, respectively, while superscript “*∗*” refers to porous materials and the subscript “*s*” refers to dense materials. *C*_1_ and *C*_2_ are dimensionless constants that depend on the type of the material, while *m*_1_ and *m*_2_ are exponentials that depend on the type of porosity (open- or closed-cell foams). According to (1) that was proposed by Ashby et al. [59], strength and elastic modulus of porous materials are increased as porosity is decreased.

However, in such theoretical models critical factors such as type of phases, grain size, and microstructure are not taken into consideration [59]. Thus, stress concentration and deformation characteristics are neglected [60, 61]. On the contrary, finite element models are more reliably used for the simulation of the mechanical behavior of porous implants [56, 62]. More specifically, Niu et al. developed a two-scale model that describes the mechanical behavior of porous materials and takes into consideration both the macro- and the microporous structure [63].

As aforementioned, porosity affects the mechanical properties of porous implants [59, 64, 65]. According to Jha et al., Young's modulus decreases linearly when density is decreased [66]. Consequently, it is possible to manufacture Ti-implants with elastic modulus comparable to that of human cortical bone (6.9–25.0 GPa) [39, 41]. Xue et al. fabricated porous implants with 27% porosity and pore size that ranged between 100 and 800 *μ*m, using laser engineered net shaping (LENS) method [51]. They found values of elastic modulus and mechanical strength that ranged between 2.6–44 GPa and 24–463 MPa, respectively [51]. Furthermore, Chen et al. fabricated cp-Ti samples with two different porosities (64% and 76%) using cp-Ti powder and H_2_O_2_ as foaming reagent [55]. The values for compressive strength and modulus of elasticity for the two different porosities were shown in Table 1.

Similarly, in another study, the modulus of elasticity of a Shape Memory Alloy (SMA) was investigated [67]. Rod-shaped porous NiTi specimens with different porosities were fabricated via powder metallurgy technique. As the porosity increased from 35.5% to 42.1%, Young's modulus decreased from 8 GPa to 6 GPa [67].

Pore size has been also investigated as a factor influencing the mechanical properties [68–70]. Tuncer et al. conducted compressive tests on cp-Ti with different porosities between 35% and 75% and pores size between 150 *μ*m and 1700 *μ*m [69]. They found that both the elastic modulus and the compressive strength were increased from 3.8 GPa to 6.1 GPa and from 43 MPa to 87 MPa, respectively, when the size of macropores was increased from 150 *μ*m to 1700 *μ*m [69]. Furthermore, pore randomization (uniform or of different sizes, orientation, and shape of pores) may increase stress concentrations in a microstructural level and provoke early localized plastic deformation [71].

Stress concentrations inside the pores of Ti-implants can be reduced by silanization (2.0% of 3-methacryloxypropyltrimethoxysilane) of the walls of the pores and subsequent filling of the pores with poly-methylmethacrylate (PMMA) [72]. This procedure results in higher tensile strengths (50–250 MPa, depending on the porosity and pore size) due to adhesion of the PMMA to the titanium pore walls leading to reduced stress concentrations [72]. Thus, it is possible to manufacture porous Ti-implants with improved tensile strength, while maintaining the elastic modulus in the same low levels (15–60 GPa, depending on the porosity and pore size) [72]. PMMA presents a significantly lower elastic modulus (2–4 GPa) than titanium. Eventually, the Young modulus of the PMMA-Ti complex remains low, despite the increase in tensile strength [72]. However, there are still concerns about the toxicity of the residual methylmethacrylate (MMA) monomer that remains after polymerization [73, 74]. It can be concluded that porous materials have great advantages, such as a low modulus of elasticity and osteoconductive properties, but there are also some disadvantages. Much progress should be made to improve their mechanical properties, so that they will become suitable biomaterials for load bearing orthopedic and dental implants.

Different methods have been proposed for manufacturing cp-Ti or Ti-alloy porous implants. The fabrication method directly affects the porosity, pore size, orientation and geometry, presence of impurities, and contamination of the biomaterial. Some of the fabrication techniques are as follows:

- loose sintering powder [75],
- space holder method [50, 76, 77],
- spark plasma sintering or field assisted consolidation technique [78, 79],
- microwave sintering [80],
- metal injection molding [81],
- capsule free hot isostatic pressing [82, 83],
- solid state isothermal foaming technique [84],
- freeze casting [85, 86] and reverse freeze casting [87],
- combustion synthesis [88],
- slip casting [89],
- gel casting [90],
- slurry foaming [91],
- entangled metallic wire materials [92],
- rapid prototyping techniques: selective laser melting (SLM) [93], selective electron beam melting (SEBM) [94], and laser engineered net shaping (LENS) [95].

### 2.2. *β*-Phase Ti-Alloys

Titanium is an allotropic element, which means that it exists in more than one crystallographic form [5]. At room temperature, the crystal structure of titanium is hexagonal closed-packed (hcp, *α*-phase), whereas, at 888°C, this structure transforms to body-centered cubic (bcc, *β*-phase) [5]. The transformation temperature (beta transus) is defined as the lowest equilibrium temperature at which the material is 100% beta and is strongly dependent on [5]

- the interstitial elements oxygen, nitrogen, and carbon (alpha stabilizers) which raise the transus temperature;
- hydrogen (beta stabilizer), which lowers the transformation temperature;
- metallic impurity or alloying elements which can either raise or lower the transformation temperature.

Alloying elements can be generally classified as alpha or beta stabilizers [5]. Alpha stabilizers, for example, aluminium, oxygen, and nitrogen, favor the *α*-phase of Ti within the alloy by rising the transformation temperature. Beta stabilizers, such as vanadium, tantalum, niobium, molybdenum, nickel, chromium, copper, and iron, result in stability of the beta phase at lower temperatures [5]. Hafnium and zirconium are unique in that they are isomorphous with both the alpha and beta phases. Hence, it is common to classify the Ti-alloys into four categories, referring to the phases that predominate within the alloy [5]:

- Alpha.
- Near-alpha.
- Alpha-beta.
- Beta.

In the titanium biomaterials currently used, the crystallographic structure of titanium is alpha phase (cp-Ti) or alpha-beta phase (Ti-6Al-4V, Ti-6Al-4Nb) [42].

The w/v percentage of the beta stabilizer into the Ti-alloy is crucial in order to obtain the bcc crystallographic structure [5]. In one study, the structure-property relationship of cast Ti-Nb alloys was tested [96]. The authors after using X-ray diffraction concluded that alloys containing 15% w/v or less niobium are dominated by hexagonal alpha phases, whereas in alloys with 27.5% w/v niobium metastable beta phase starts to be retained. When niobium contents become 30% w/v or higher the beta phase was almost entirely retained.

Moreover, *β*-phase Ti alloys seem to be a very promising material for biomedical applications, due to their low elastic modulus and increased corrosion resistance [97, 98]. The elastic moduli of different *β*-phase Ti alloys are listed in Table 2. The elastic behavior of Ti-alloys with *β*-phase stabilizer strongly depends on the concentrations of the stabilizers inside the alloy [99–101]. Sakaguchi et al. investigated the effect of Ta content on the elastic modulus of a Ti-30Nb-*X*Ta-5Zr alloy (*X*_*O*_ = 0, *X*_1_ = 5, *X*_2_ = 10, *X*_3_ = 15, *X*_4_ = 20) [101]. They reported that for Ta contents below 10% w/v the alloy displays a Stress Induced Martensite (SIM) behavior, while its microstructure consists of *β*- and *ω*-phases [101]. As the content of Ta increases to 10% Young's modulus decreases and the alloy becomes more elastic [101]. When the Ta content exceeds 15% w/v, the *β*-phase predominates within the alloy; thus the elastic modulus increases towards the elastic modulus of pure Ta (181 GPa) [101]. On the contrary, Zhou et al. reported that Young's modulus of the Ti*X*Ta alloy shows its maximum decrease (65 GPa) at *X* = 30% w/v, then peaks at *X* = 50% w/v (90 GPa), and finally decreases again (65 GPa) at *X* = 70% w/v [100]. Additionally, Correa et al. investigated the effect of Zr content on Young's modulus of Ti*X*Zr alloys (*X* = 0, *X* = 5, *X* = 10, *X* = 15) [102]. The results indicated that the elasticity of the alloy is increased at a Zr content of 5% w/v, whereas higher concentrations of Zr lead to an increase of the elastic modulus of the alloy [102].

The corrosion resistance of *β*-phase Ti alloys has also been investigated extensively. Ribeiro et al. conducted electrochemical impedance spectroscopy (EIS) tests to assess the corrosion behavior of Ti-35Nb-5Zr and Ti-35Nb-10Zr, using Ti-6Al-4V as control [103]. The mean passive current densities (*i*_pass_) of the 2 alloys (*i*_pass (Ti-35Nb-5Zr)_ = 6.28 ± 0.34 *μ*A/cm^2^ and *i*_pass (Ti-35Nb-10Zr)_ = 11.90 ± 4.11 *μ*A/cm^2^) were comparable (*p* > 0.05) with this of Ti-6Al-4V (*i*_pass (Ti-6Al-4V)_ = 7.29 ± 0.85 *μ*A/cm^2^). The mean corrosion potentials (*E*_(*i*=0)_) between the three alloys (*E*_(*i*=0) Ti-35Nb-5Zr_ = 0.276 ± 0.058 V, *E*_(*i*=0) Ti-35Nb-10Zr_ = 0.349 ± 0.060 V, *E*_(*i*=0) Ti-6Al-4V_ = 0.286 ± 0.015 V) were not statistically significant (*p* > 0.05) [103].

However, the manufacturing processes appear to be very important to the corrosion resistance of the material [104, 105]. Raducanu et al. compared the corrosion resistance between an as-cast Ti-10Zr-5Nb-5Ta alloy and an Accumulative Role Bonding (ARB) processed Ti-10Zr-5Nb-5Ta alloy in Ringer's solution (pH = 6.9, 37°C) [104]. After cyclic potentiodynamic polarization, they concluded that the ARB-processed alloy exhibits better corrosion resistance (*i* = 0.142 *μ*A/cm^2^, *E*_corr_ = 0.450 V) than the as-cast (*i* = 0.230 *μ*A/cm^2^, *E*_corr_ = 0.450 V) [104]. Furthermore, Gill et al. investigated the corrosion behavior of Ti-30Ta manufactured by two different fabrication methods, powder metallurgy (PM) and ARC-melting (ARC) [105]. The cyclic potentiodynamic corrosion curve of the ARC manufactured Ti-30Ta exhibited a clockwise loop with hysteresis, related to less corrosion resistance when compared to PD [105].

Among the beta stabilizers reported, niobium, zirconium, and tantalum present favorable biocompatibility results. In one study, niobium and zirconium exhibited excellent biocompatibility, while tantalum exhibited medium biocompatibility when tested in cell cultures of murine calvaria osteoblast-like cells (MC3T3-E1) [42]. In another study, TI-25Nb alloy samples with different percentages of porosity were inserted for 3 h, 24 h, and 72 h, in a rabbit bone marrow mesenchymal stem cell (BMMSC) incubator, at 37°C, 5% CO_2_, and 100% relative humidity [106]. After fluorescent microscopy and SEM observations it was concluded that Ti-25Nb alloys show good biocompatibility regardless of the percentage porosity. Similarly, in another in vitro study, De Andrade et al. assessed different parameters of osteogenesis of dense and porous cp-Ti and Ti-35Nb samples in rat calvaria-derived cells [107]. The findings indicated that cell numbers were higher in the cp-Ti sample at 3 days of cultivation (*p* < 0.05) [107]. However, at 7 days, cell numbers were not statistically significant between the two materials [107]. MTT assay revealed that cell viability was not affected by the type of the material, whereas, after 14 days, dense Ti-35Nb samples exhibited the highest alkaline phosphatase activity (ALP) (*p* < 0.05) [107]. In addition, in an in vivo study, Ti-50Zr alloy samples were tested after 8 months of implantation inside spleens of female F344/DuCrj rats. After hematological and histological analyses, it was outlined that the alloy presents better biocompatibility than cp-Ti [108]. Sista et al. compared in vitro the biological behavior of Ti-50Nb, Ti-50Zr, and cp-Ti using mouse osteoblast cell cultures (MC3T3-E1) [109]. After the first 4 hours of plating there were statistically significant differences (*p* < 0.05) between cell adhesion on Ti-50Zr (35%) and cp-Ti (27%) and Ti-50Nb (27%) [109]. However, regarding the spreading of cells there were similar findings on all surfaces at 8 hours of plating [109]. Data from cell viability suggests that after 24 hours the survival of cells on the Ti-50Zr surface was 70%, while the respective value for cp-Ti and Ti-50Nb was 50% (*p* < 0.05) [109]. Cell numbers were 17.5 × 10^3^ for the Ti-50Zr and 17 × 10^3^ for cp-Ti, which were significantly (*p* < 0.05) higher than those of Ti-50Nb (11 × 10^3^) [109]. Similarly, ALP activity at 7 days was significantly higher (*p* < 0.05) for cp-Ti and Ti-50Zr (~0.155 (IU/L)/ug protein) than for Ti-50Nb (0.07 (IU/L)/ug protein) [109].

### 2.3. Titanium Bulk Glasses

Metals and metal alloys are characterized by a microcrystalline structure. On the contrary, bulk metallic glasses (BMGs) are metallic materials with noncrystalline structure [110]. The first BMG was produced by Klement et al. in 1960 from an Au_75_Si_25_ alloy [111]. Since then, BMGs have been widely investigated, due to their excellent mechanical properties, such as superior strength, high corrosion fatigue, wear resistance, and low modulus of elasticity [112, 113].

Inoue expressed three empirical rules to describe the glass forming ability (GFA) of the BMGs [114, 115]. Firstly, the glass-like structure of these materials is a result of solidification with an extremely high cooling rate that does not allow for the formation of the typical crystal nucleation of the alloy [110]. Furthermore, in order to achieve high glass forming ability (GFA) the alloy system should be comprised of three or more alloying elements and, thirdly, their atomic size ratios should exceed 10% [110].

Over the last 25 years many Ti-based BMGs have been fabricated based on Ti-Ni-Cu [116–121], Ti-Zr-Be [122–124], and Ti-Zr-Cu-Ni [125–128]. However, those Ti-based BMGs cannot be used for biomedical applications because of the cytotoxicity of Ni- [129] and Be- elements [130]. This fact has led to the development of ternary Ti-Zr-Cu-Pd with Pd instead of Ni- and Be- [131, 132]. Among them, Ti_40_Zr_10_Cu_36_Pd_14_, with compressive strength of 1950 MPa and Young's modulus of 82 GPa, presents the higher glass forming ability [131, 133].

Zhu et al. reported that substituting Cu by Sn at 2% in Ti-Zr-Cu-Pd BMGs improves the GFA of the alloy [134]. Furthermore, Ti-Zr-Cu-Pd BMGs with minor additions of 2% Sn present a compressive strength of 2000 MPa [135]. Oak et al. investigated the mechanical behavior of Ti_45_Zr_10_Pd_10_Cu_31_Sn_4_ BMG and reported a compressive strength of about 1970 MPa, Vickers's hardness of 650 Hv, and Young's modulus of 95 GPa [136]. Moreover, this BMG alloy presents a crystallization temperature of 681 K and a glass formation temperature of 737 K [136]. Consequently, it displays a wide supercooled liquid region (Δ*T*_*X*_) of 56 K, which may allow its shaping when heated [136]. Additionally, Qin et al. tested the corrosion rate of Ti_45_Zr_10_Pd_10_Cu_31_Sn_4_ BMG, in terms of weight loss after immersion in a 1 N HCl solution (room temperature ≈ 298 K) [137]. It was reported that the corrosion rate of this Ti-based BMG was 0.046 mm year^−1^, much lower than that of stainless steel 0.28 mm year^−1^ [137].

Furthermore, Pang et al. performed minor additions of 2% Ag, an element with antimicrobial effect and enhanced GFA [138]. They developed a Ti_47_Cu_38_Zr_7.5_Fe_2.5_Sn_2_Si_1_Ag_2_ BMG with compressive strength, specific strength, Young's modulus, and Vickers' microhardness of 2080 MPa, 3.2 × 105 N m/kg, 100 GPa, and 588 Hv, respectively [138]. Its glass transition temperature (*T*_*G*_ = 641 K) and crystallization temperature (*T*_*X*_ = 693 K) allow for a wide supercooled liquid region (Δ*T*_*X*_) of 52 K [138]. Similarly, Wang et al. investigated the effect of different Ag additions in the Ti_46_Cu_31.5−*x*_Zr_11.5_Co_3_Si_1_Ag_*x*_ (*x* = 0,1, 2,3, 4,5 at%) [139]. They indicated that when *x* = 4% the structure is fully glassy and does not present any crystalline phase. They concluded that the GFA is enhanced by the addition of Ag because the difference between the melting temperature and the crystallization temperature is increased [139].

Other elements that were investigated for minor addition into Ti-based BMGs are Si [140], Nb [141], and noble elements, such as Au and Pt [142]. According to Qin et al., the addition of 1% of noble alloys led to the development of a Ti_40_Zr_10_Cu_36_Pd_14_M_*x*_ BMG with high yield strength (2000 MPa), low Young's modulus, and improved plastic strain (1.5–10%) [142]. Furthermore, the addition of 1% Si enhanced the GFA of Ti_40_Zr_10_Cu_36_Pd_14_, due to the increase of its Δ*T*_*X*_ (60 *Κ*) [140]. A very promising element for addition to Ti-Zr-Cu-Pd BMGs appears to be Nb [141]. In a study conducted by Qin et al., different contents of Nb in Ti-Zr-Cu-Pd BMGs were investigated [141]. It was concluded that the addition of 3% Nb leads to the development of a BMG with superior mechanical properties (yield strength: 2050 MPa, Young's modulus: 80 GPa, and plastic strain: 6.5%). Moreover, according to Fornell et al., the addition of 3% Nb in the Ti-Zr-Cu-Pd BMGs results in superior corrosion resistance (corrosion density: 3.77 × 10^−6 ^A/cm^2^, corrosion potential: 0.007 V, and pitting potential: 0.412 V) [143].

Recently, Wang et al. develop another Ti-Cu-Hf-Si BMG, based on a binary-eutectic rule that allows the prediction of GFA in relation to the composition [144]. The rule is based on (2) to predict the composition Cam of the BMG [145]:

(2) CamαTi57Cu42+βCu56.4Hf43.6+γTi86.5Si13.5,αΔHTiCuβΔHCuHf=γΔHTiSi,

where *α*, *β*, and *γ* are coefficients for the three main units and Δ*H*_(TiCu)_, Δ*H*_(CuHf)_, and Δ*H*_(TiSi)_ are the mixing heats of the clusters, −9 kJ/mol, −17 kJ/mol, and −66 kJ/mol, respectively [145]. From (2) arises the fact that *α* = 0.6, *β* = 0.318, and *γ* = 0.082. So the Cam becomes Ti_41.3_Cu_43.7_Hf_13.9_Si_1.1_ [144]. This BMG exhibits ultimate strength of 1685 MPa, Young's modulus of 95 GPa, and supercooled region (Δ*T*_*X*_) of 40°C [144]. Furthermore, its corrosion potentials in NaCl and Hank's solution are −0.164 V and −0.624 V, respectively, indicating a good corrosion resistance comparable with that of Ti-6Al-4V [144].

Concerning the biocompatibility of Ti-based BMGs, Oak et al. tested the cytotoxicity of Ti_45_Zr_10_Cu_10_Pd_31_Sn_4_ in vitro, using osteoblast cell cultures (SaOS2) in 36.85°C for 8 days [146]. They reported that the content of Cu in the BMG was not enough to cause cytotoxicity, due to the biochemical corrosion after immersion to Phosphate Buffered Solution (PBS) and 1% lactic acid solution [146]. Similarly, Pang et al. investigated the cytotoxicity of the Ti_47_Cu_38_Zr_7.5_Fe_2.5_Sn_2_Si_1_Ag_2_ BMG using cultures of the MC3T3-E1 cell-line for 3 days [138]. The Ti-6Al-4V alloy was used as a control. They reported that the biocompatibility of the BMG is comparable with that of a Ti-6Al-4V alloy. SEM observations on the surface of the Ti_47_Cu_38_Zr_7.5_Fe_2.5_Sn_2_Si_1_Ag_2_ BMG revealed overlapped layers of cells, connected with cytoplasmic elongations, while on the surface of the Ti-6Al-4V samples there was one layer of polygonal cells [138]. Wang et al. performed direct and indirect cytotoxicity tests in order to investigate the biocompatibility of Ti_41.5_Zr_2.5_Hf_5_Cu_37.5_Ni_7.5_Si_1_Sn_5_ BMG (TZHCNSS) [147]. They used murine fibroblast cultures (1929 cells and NIH3T3 cells) and cp-Ti as control. However, the results indicated proliferation rates of 60% for 1929 cells and 55–65% for NIH3T3 cells at 4 days [147]. Despite this low cell viability in vitro, which was attributed to Cu content, the in vivo results were more promising [147]. After 1 month of implantation in the mandibles of 6 dogs, light microscopy (100x magnification) observations revealed good osteointegration. The difference between in vitro and in vivo tests was attributed to the fact that in a living organism the metabolism does not allow for the accumulation of Cu ions [147].

Moreover, not all surface treatments are feasible on BMGs, because only low temperature processes can be performed without compromising the mechanical properties of these materials [148–150]. Qin et al. investigated the ability of rod-shaped Ti_40_Zr_10_Cu_36_Pd_14_ BMG to form an apatite layer after acidic (5% HCl + 30% HNO_3_ for 10 s at room temperature), alkali, and heat treatment (5 M NaCl at 60°C for 24 h) and immersion in simulated body fluids (SBF) [148]. They highlighted that despite the fact that the BMG became porous there was no apatite layer formation [148]. For this reason, they created a pure-titanium layer, using Ti sputtering technique before the alkali and heat treatments. After X-ray energy dispersive spectroscopy (EDS), a sodium titanate layer was observed on the surface of the BMG. Following 15 days of immersion in SBF a CaP apatite layer of 300 nm thickness was developed on the surface of the Ti-treated Ti_40_Zr_10_Cu_36_Pd_14_ BMG [148]. The apatite formation mechanism is further explained in the following chapter for surface treatments. However, the bonding strength between the Ti-coating and the BMG should be further investigated [148]. Additionally, the apatite formation ability of a Ti_42_Hf_11_Pd_11_Cu_36_ BMG was induced after anodic oxidation by potentiostatic polarization at 25°C in a 1 M NaOH solution [150]. In another study two other coatings of TiN and (Ti, Al)N on Ti_40_Zr_10_Cu_36_Pd_14_ BMG were developed by magnetron sputtering (PVD) [149]. The porosities of the Ti_40_Zr_10_Cu_36_Pd_14_ BMG with the two different coatings are 6.4% and 2.7% for TiN- and (Ti, Al)N-, respectively [149]. The corrosion passive current density was 8.8 × 10^−4 ^A/m^2^ and 8.4 × 10^−4 ^A/m^2^ for TiN- and (Ti, Al)N-coatings, respectively, when calculated by polarization curves in Hank's solution [149]. Those values are much lower than that of the BMG substrate (3.5 × 10^−3 ^A/m^2^), showing that these coatings probably decrease the overall passive current density of the material, increasing its corrosion resistance [149]. The corrosion resistance is further enhanced because of the increase of the corrosion potential at 0.02 V for the TiN-coated BMG and at 0.05 V for the (Ti, Al)N-coated BMG [149].

## 3. New Surface Treatment Modifications

### 3.1. Surface Modifications to Improve the Mechanical Properties of the Implant

#### 3.1.1. Anodic Oxidation

Anodic oxidation is an accelerated electrochemical oxidation process based on electrode reactions and leading to the formation of an oxide film on the anode metal surface [152]. Titanium is coated by an oxide surface layer of 1.5–10 nm thickness that is formed naturally on the titanium surface on the exposure to air at room temperature [153]. Consequently, anodic oxidation of titanium surface results in the production of a thicker oxide film than that formed spontaneously at the metal surface. It should be mentioned that the oxide film has very important role in the implant's biocompatibility since it is this surface rather than the main body of the titanium implant that comes in direct contact with the bone tissue [154]. Based on this fact, many surface modifications have been investigated so as to enhance the properties of this thin oxide layer [155]. Among the various techniques, the anodic oxidation is a well-established and promising method, since it can produce different types of oxide films on titanium surfaces. The thickening of the oxide film obtained by the anodic oxidation may contribute to the increase of the corrosion and wear resistance, as well as to the improvement of the adhesion and bonding [152].

The properties of the formed oxide layer depend on the process parameters, such as the anode voltage and the electrolyte composition. Regarding the applied voltage, high voltages produce thick and porous oxide films, while low voltages produce thin and compact films [154]. As for the electrolyte, different acids such as H_2_SO_4_, H_3_PO_4_, and acetic acid, neutral salts, and alkaline solutions can be used for the titanium anodization [156, 157]. It has been reported that the alkaline electrolytes such as calcium hydroxide and sodium hydroxide revealed a relatively lower ability of anodic oxide formation than the acidic ones [156].

The titanium oxide layer shows three different crystalline forms: anatase (tetragonal), rutile (tetragonal), and brookite (orthorhombic). It has been reported that the anatase and rutile structures of the titanium oxide layer have the ability to form hydroxyapatite, a bioactive material that can induce bioactive bonding with the surrounding bone enhancing the osseointegration [158–160]. Yang et al. have demonstrated that the anodic oxidation in H_2_SO_4_ solution combined with heat treatment at 600°C for 1 h led to the apatite formation due to the increasing of the anatase and rutile structures in the titanium oxide layer [159]. It has been revealed that the anodic oxidation treatment led to the increase of the corrosion resistance of titanium implants [161]. Leinenbach and Eifler investigated the influence of the oxidation treatment on the fatigue behavior of the titanium and found that the oxide film obtained by the anodic oxidation could withstand higher stress amplitudes (325 Mpa) and higher plastic strains (before the first surface damage) than the oxide films produced by thermal oxidation (275 MPa).

#### 3.1.2. Chemical Vapor Deposition (CVD)

Chemical vapor deposition is a process resulting in the deposition of a solid material on a substrate's surface from the chemical reaction between gaseous precursors and the heated surface of the metal substrate [162, 163]. A gas delivery system supplies the reactor chamber of the CVD apparatus with chemical gases which come into contact with a heated substrate in order to react and decompose forming a solid phase that coats the substrate. The heating of the substrate may be accomplished by tube furnaces, halogen lamps, induction heating lasers, or UV light. Furthermore, the gaseous precursors can be halides, hydrides, and various metal compounds such as carbonyls and alkoxides. During this process, chemical by-products are produced that are removed from the reactor chamber along with the unreacted precursor gases via an exhaust and a vacuum system [152, 163]. Various types of the CVD processes have been used such as the atmospheric pressure CVD, the low pressure CVD, the metal-organic CVD, the plasma-assisted CVD, the laser CVD, the photochemical CVD, the chemical vapour infiltration, and the chemical beam epitaxy [163].

Among the various applications of the CVD [162], the formation of a solid coating on the metal surface resistant to the corrosion and wear is an attractive method for the preparation of implants surface intended for a highly corrosive environment like that of the human body [164]. It has been demonstrated that the CVP process of titanium implants results in the enhancement of the wear and corrosion resistance of these materials. Furthermore, many authors found that the diamond-like-carbon (DLC, an amorphous form of carbon) coating produced by various CVD methods on the titanium surfaces has enhanced mechanical properties, such as wear and corrosion resistance along with a good biocompatibility [165–168]. However, it should be mentioned that the thermal expansion of the diamond film is highly different from the thermal expansion of the titanium leading to a poor film adhesion on the titanium substrate [152]. It has been demonstrated that this drawback can be overcome by the use of an intermediate layer in order to improve the adhesion of the DLC coating [165, 169, 170]. Specifically, Kim et al. prepared DLC-coated Ti alloys with the incorporation of an amorphous silicon intermediate layer using the plasma-assisted CVD technique and found an improvement in the corrosion resistance in a simulated corrosive environment of the body fluid by a 0.89% NaCl solution [166]. This finding is in agreement with the result of another study, which demonstrated that the wear resistance of a 1 *μ*m thick DLC layer was statistically significant higher than that of the noncoated titanium surfaces [165]. Moreover, the anticorrosion nature of the silicon layer incorporated to titanium surfaces has been also supported by other studies [167, 171]. Lastly, except from the DLC coating, the formation of titanium carbide layer on titanium surface by an ion-enhanced triode plasma CVD led to high abrasion resistance encouraging the use of this layer as abrasion resistant implant material [172].

### 3.2. Surface Modifications to Induce Bioactivity, Cell Growth, and Osseointegration

#### 3.2.1. Sandblasting/Grit-Blasting, Acid-Etching: Formation of Porous Surface

The surface characteristics of the implants affect the behavior of the surrounding bone and consequently the implant's osseointegration process. Various studies have reported that the implants with rough surfaces demonstrated better behavior and higher survival rates compared with the machined ones [173–175]. Specifically, Pinholt found that the survival rate of rough implants was 98%, while that of machined implants was 81%, in a follow-up period of 20 to 27 months [174]. Furthermore, Gotfredsen et al. revealed that implants blasted with titanium-dioxide-particles showed a better anchorage and bone-implant contact than the machined implants [175]. The superiority of rough implants over the machined ones regarding bone formation is based on the surface microtopography modification and the alteration of the surface energy which leads to proteins and blood components absorption enhancing the cell attachment and implant osseointegration [176].

Based on this finding, various surface modifications have been tried to produce micro-rough titanium surfaces, including sandblasting, acid-etching, and surface chemistry alterations, in order to enhance the osseointegration process of these implants. The blasting process is based on abrasive particles (i.e., alumina, corundum, rutile, and hydroxyapatite) forced against the implant's surface. Regarding the acid-etching process of titanium surfaces, HCl, H_2_SO_4_, and HF are used as acids, since they are able to react with the oxide layer formed on the titanium surface [177]. As for the surface chemistry, ionic interactions, protein absorption, and cellular activity at the implant surface are altered, resulting in modifications of biologic events such as the osseointegration [178].

Specifically, Abdel-Haq et al. found that the chemically modified sand-blasted acid-etched implants achieved a higher bone-to-implant contact compared with the standard sand-blasted acid-etched implants in the early weeks of healing, while no statistically significant difference was found in the bone contact between the 2 types of implants after 6 weeks of healing [179]. It should be mentioned that the surface chemical alteration by nitrogen rinsing and storage in a NaCl solution results in increased wettability and hydrophilicity of the titanium surface [179–181]. Various studies have reported that the hydrophilic nature of implants which were modified by sand-blasting and acid-etching improved their osseointegration when compared with unmodified rough implants [180–183]. Similarly, Patel et al. supported the significance of the wettability and hydrophilicity of micro-rough implants in the cellular attachment and subsequently in their effective osseointegration. They mentioned that the hydrophilicity of the micro-rough titanium implants increased after each one of the following treatment procedures: (a) deionized water rinse followed by nitrogen drying, (b) sonication in methanol, (c) deposition of a 10 nm thick TiO_2_ film, and finally (d) water wash and nitrogen drying of the rough titanium samples covered with the TiO_2_ layer [184]. Also, the same authors stated that the high hydrophilicity is attributed to the removal of the inorganic and organic surface contaminants by the cleaning treatment. Finally, a prospective study by Karabuda et al. demonstrated that the marginal bone loss difference was statistically significant between the standard and modified sand-blasted, acid-etched implants after 15 months of evaluation, while the difference of the survival rates between the 2 types of implants was not statistically significant [185].

#### 3.2.2. Alkaline Treatment: Coating CaP

An alkali-treated Ti-surface is negatively charged [186]. Consequently, in acellular simulated biofluids (SBF), it tends to absorb the positively charged calcium ions [186, 187]. As the accumulation of calcium progresses, the surface becomes more positively charged, leading to phosphate ion absorption and thereafter apatite formation [186, 187]. This procedure was experimentally confirmed by X-ray photoelectron spectroscopy for different time intervals [188, 189].

Furthermore, for the apatite layer formation, heat treatment after alkali solution exposure is essential. More specifically, when a Ti-surface is exposed to an alkali solution (i.e., NaOH) sodium hydrogen titanate is formed [190]. By heat treatment, sodium hydrogen titanate transforms to sodium titanate (Na_2_Ti_3_O_7_), which exchanges Na^+^ ions for H_3_O^+^ in SBF [190].

In an in vivo study, Yan et al. exposed Ti-rectangular specimens to alkaline solution of 5 M NaOH at 60°C for 24 h and then heat treated them at 600°C for 1 hour [191]. Afterwards the specimens were inserted into 8 tibias of 4 rabbits. Eight weeks after implantation, the surrounding woven and lamellar newly formed bone was observed in direct contact with an apatite layer on the surface of the specimens. Similarly, in another animal study, a Ti-metal rod was exposed in 5 M NaOH solution at 60°C for 24 h and heat treated to 600°C for 1 h. Four weeks after implantation into a rabbit femoral condyle, bone islands were present even in the deepest part of the pores on the surface of the specimen [192]. Tsukanaka et al. investigated the proliferation and differentiation of osteoblasts around alkali and heat treated cp-Ti culture plates—in 5 M NaOH solution at 60°C for 24 h and heat treated to 600°C for 1 h—versus nontreated cp-Ti plates, using fluorescent primary osteoblasts [193]. It was reported that the onset of differentiation on the surface treated plates was accelerated. However, once the procedure had started no important differences between the two groups were identified. Nevertheless, SEM observations have disclosed that the osteoblasts on the surface treated plates were small and round, whereas the osteoblasts on the nontreated surface group were larger and flat [193].

Concerning Ti-alloys, alkali and heat treatments have been attempted in Ti-6Al-4V [194–196], Ti-6Al-2Nb-Ta [194, 196], and Ti-15Mo-5Zr-3Al alloys [194, 196]. Apatite formation in SBF and bone bonding were achieved on the surfaces in all above mentioned alloys and are attributed to specific alloying elements (i.e., Al, V, and Mo), whereas, in other Ti-alloys, which contain Ta, Zr, and Nb, like TNZT alloys (Ti-Nb-Zr-Ta), the sodium release is inhibited and apatite formation is suppressed [194, 196]. Another disadvantage of the alkali-heat-treated titanium surfaces is that the apatite formation is sensitive to even small amount of Ca ions [197].

The above problems have resulted in the replacement of sodium titanate with calcium titanate [198–200]. After the exposure in a NaOH solution, the Ti-alloy was soaked in a 100 Mm CaCl_2_ solution at 40°C for 24 h. This step allows the Na^+^ ions to be replaced by Ca^2+^ ions and, subsequently, the sodium titanate to be replaced by calcium titanate. In SBF, the Ca^2+^ ions are replaced via exchange with H_3_O^+^ ions, resulting in the formation of TiOH on the Ti-metal surface. This procedure progressively makes the surface more negatively charged and combines with the positively charged Ca^2+^ ions that had been released. Then, negatively charged phosphate ions are attracted by the Ca^2+^ ions, resulting in a crystalline apatite [198]. This treatment has allowed apatite formation on new Ti-alloys such as Ti-15Zr-4Nb-4Ta [199, 201], Ti-29Nb-13Zr-4.6Ta [201], and Ti-35Nb-2Ta-3Zr-0.3O [200, 202].

Concerning clinical applications of alkaline and heat treatments, a total of 70 hip arthroplasties were performed in 58 patients (mean age 51.7 years), using orthopedic implants made of Ti-6Al-2Nb-1Ta alkaline and heat treated [203]. In a mean 10-year follow-up the overall survival rate was 98% (CI 95%) [204]. Two implants were retrieved after failing due to deep infection and periprosthetic femoral fracture—at 2 weeks and 8 years after implantation—and underwent histologic examination, which revealed newly grown bone, even on the implant, which was retrieved as early as 2 weeks after implantation [204]. Additionally, 5 spinal fusion devices, made of alkaline and heat treated cp-Ti, were applied to 5 patients with a very successful outcome and a rapid recovery [205]. Future applications of apatite layer include the utilization of these layers as carriers for drugs [206], such as bisphosphonates, growth factors [207], and DNA [208].

#### 3.2.3. Acid Treatment: Coating CaP

Furthermore, acid treatment leads to a positively charged titanium surface that has an affinity to absorb negatively charged phosphate ions in SBF. Afterwards, the accumulation of phosphate ions, which makes the titanium surface progressively more negatively charged, provokes the absorption of calcium ions. This procedure gradually leads to apatite formation on the titanium surface, as was confirmed by X-ray photoelectron spectroscopy [186, 209].

It seems that heat treatment after exposure to acidic solution enhances bone formation [210, 211]. In one animal study, two Ti-metal specimens that had been exposed to a strong acidic solution (66.3% w/w H_2_SO_4_ solution and 10.6% w/w HCl solution in a 1 : 1 weight ratio) for 1 h and heat treated at 600°C for another 1 h were implanted into rabbit tibias [210]. Four weeks after, newly formed bone with parallel collagen fibers in direct contact with one specimen was observed and, eight weeks after, bone remodeling had occurred on the surface of the second specimen. On the contrary, in specimens that had been exposed to pure water instead of acidic solution prior to heat treatment or had not been heat treated at all, fibrous tissue encapsulation was observed [210]. These findings are in agreement with another in vivo study, where Ti-specimens were exposed to the same acidic solution (H_2_SO_4_/HCl) for 30 min and heat treated at 600°C for 1 h [211]. The specimens were inserted into a dorsal muscle of a beagle dog and after 12 months newly formed ectopic bone was observed on its porous and charged surface (zeta potential: 8.0 (±2.0) mV). On the contrary, in the same study, specimens that had not been heat treated after acidic exposure or that had been exposed to pure water prior to heat treatment showed no bone formation within 12 months, almost zero surface charge (zeta potential: ~0 mV and −2.1 (±3.1) mV, resp.), and no surface apatite formation after being embedded to SBF [211].

Things become more complicated when it comes to Ti-alloys [209]. Alloying elements form other oxides—apart from Ti-oxides—on the alloy surface, limiting the capacity of apatite formation in SBF. However, in the case of Ti-15Zr-4Nb-4Ta, exposure to NaOH solution, prior to HCl solution and heat treatment, resulted in a positively charged titanium oxide layer and apatite formation in SBF [212].

Furthermore, findings from the above studies indicate that titanium exposure to acidic solution and following heat treatment has not only osteoconductive but also osteoinductive properties, due to the apatite layer formation in its surface.

#### 3.2.4. Biochemical Modification

Biochemical modification aims at utilization of cell adhesion and differentiation properties in order to achieve faster osseointegration and bone adhesion. Surface immobilization of extracellular matrix (ECM) proteins and their effects in bone stimulation have been investigated thoroughly. More specifically, attempts have been made in order to immobilize proteins [213, 214], peptides [215], and growth factors on implant surfaces [216].

Among ECM proteins, collagen I is a very promising candidate for protein immobilization [217]. The procedure includes amino groups deposition from allylamine plasma, placement in a 0.1% collagen type I solution at 37°C overnight, to initiate fibrillogenesis, and covalent linking obtained by carbodiimide (EDC) and N-hydroxysuccinimide in an aqueous solution [218]. Morra et al. have evaluated the regenerative properties of collagen I coatings, in vitro, both on osteoblast-like cell (SaOS2) cultures [218] and on human mesenchymal cell (HMC) cultures [219]. In the first study, collagen coated titanium (ColTi) and noncoated titanium (Ti) had no significant differences, concerning the growth of SaOS2 cells [218]. On the contrary, in the case of HMC, ColTi stimulated cell adhesion and density in short experimental time and the surface details were completely followed by the cell bodies [219]. The above findings suggest that osteoblast-like cells' adhesion is controlled by surface topography, whereas HMC are chemically stimulated [218, 219]. In the above-mentioned studies, covalent immobilization was used; however absorptive immobilization of collagen I has also been described [213]. In this case, the titanium samples were soaked in a 0.1% collagen type I solution for 6 h, water cleaned ultrasonically, and finally dried under vacuum [213]. Ao et al. evaluated absorptive and covalent immobilization of collagen type I on Ti-6Al-4V samples, concerning adhesion, proliferation, and differentiation of HMC [213]. Their findings suggest that covalent immobilization is superior to absorptive, regarding both the amount and stability of collagen type I [213].

Furthermore, in vivo studies have also showed successful outcomes [218, 220–222]. Morra et al. investigated bone to implant contact of 8 ColTi implants and 8 noncoated Ti implants, in the cortical bone of femur and the trabecular bone of tibia of eight adult rabbits [218]. Two weeks after implantation, bone to implant contact was sufficiently higher in ColTi implants than in the noncoated Ti implants. Additionally, Sverzut et al. implanted 12 titanium implants, surface treated with covalently immobilization of collagen type I, in the mandibles of 6 mongrel dogs [221]. Another group of 12 nontreated titanium implants was also implanted to be used as control. The animals were euthanized 3 and 8 weeks after implantation and histomorphometric, cellular, and molecular analyses were performed. Histomorphometric analyses showed no signs of inflammation or fibrous tissue formation adjacent to the implants, while surface treatment clearly affected bone to implant contact (*p* < 0.001). Cellular analysis showed no difference in the number of osteoblastic cells, but there were higher levels of alkaline phosphatase adjacent to ColTi implants. Molecular analysis indicated that RNA that was extracted from bone adjacent to ColTi implants showed higher expression of genes that encode alkaline phosphatase, runt-related transcription factor 2, osteocalcin, and bone sialoprotein [221]. In another study, Korn et al. inserted 36 screw-type cp-Ti implants in the mandibles of 6 female Berlin minipigs [220]. Of the 36 implants, 12 were noncoated, 12 were surface treated resulting in a chondroitin sulfate-containing collagen coating, and the rest of 12 were surface treated resulting in a sulfated hyaluronan-containing collagen coating. After 4- and 8-week healing periods, histologic evaluation indicated higher bone maturation in the coated implants. After the 4-week healing period chondroitin sulfate-containing collagen coated implants had statistically significant superior spongious bone to implant contact (spongious BIC) compared to sulfated hyaluronan-containing collagen coated implants (*p* < 0.05), whereas after 8 weeks of healing this difference was not statistically significant [220]. Last but not least, Sartori et al. compared ColTi coated implants versus noncoated Ti implants in 20 healthy and 20 osteopenic rats [222]. The implants were made of cp-Ti and received a hydrofluoric acid-etching treatment. The collagen was covalently immobilized onto implant surfaces. The implants were implanted into the femoral condyles of the rats and left for a healing period that ranged from 4 to 12 weeks. Each rat received a ColTi implant in the right condyle and a Ti implant in the left condyle. Histomorphometric investigation showed that the total bone to implant contact was significantly higher in the ColTi implants, compared to Ti implants in both the healthy and osteopenic rat models [222].

Lately,* short biomimetic peptides* have been immobilized on implant surfaces [215, 223]. Short peptides are used because they are cheaper and they can be more easily obtained in high purity, larger quantity, and better stability [215]. The mostly used peptides are tripeptide Arg-Gly-Asp (RGD) motifs that are derived from fibronectin and vitronectin, which play a pivotal role in cell adhesion [215]. Dettin et al. used two peptides for surface functionalization, (GRGDSP)_4_K which contains tripeptide RGD and (352–360) HPV, and investigated the properties of promoting cell adhesion of osteoblast-like cells [215]. The (313–324) HIV-I_MN_gp120 peptide was used as a control. The peptides were covalently immobilized onto the surfaces of cp-Ti (grade 2) disks. Adhesion assays were conducted on Sprague-Dawley rat osteoblasts cells. The findings indicated that both peptides result in an increase of osteoblast adhesion whereas (352–360) HPV leads to higher bone density [215].

*Growth factors* have also been immobilized onto implant surfaces to obtain a biomimetic implant behavior [216]. Seol et al. investigated a synthetic peptide which mimics BMP2. The peptide was immobilized onto titanium disk surfaces via a cross-linker SMCC. The procedure included APTES grafting, an activation of the implant surface via a 2% hexane solution (stirred for 30 min under argon bubbling). Then, the disks underwent reaction with SMCC and finally they were washed and grafted with the peptide in a 2 mg/0.5 mL phosphate buffered saline [216]. The disks were tested both in vitro, in osteoblast-like MC3T3-E1 cells, and in vivo, after implantation into the mandibles of two beagle dogs. The growth rate of the MC3T3-E1 cells in the case of the surface treated disks was significantly higher than in the untreated ones, which were used as a control [216]. Furthermore, using fluorescently labeled phalloidin, adherent microfilament bundles of actin were observed to the surface of the peptide Ti-disks, whereas no evidence of such attachment was noticed on Ti-disks. Regarding the implants that were retrieved from the mandibles of the dogs, the histological analysis showed higher bone maturation in the peptide Ti-disks with thicker trabeculae, indicating a faster bone maturation [216].

### 3.3. Surface Modifications with Antibacterial Effects

#### 3.3.1. Antibiotic and Nonantibiotic Organic Coatings

Antibiotics with broad antibacterial spectra, such as gentamicin, cephalothin, carbenicillin, amoxicillin, cefamandole, metronidazole, simvastatin, tobramycin, and vancomycin, have been incorporated in coatings of bone implants. Calcium phosphate and carbonated hydroxyapatite have been used in in vitro studies as carriers of vancomycin and tobramycin to minimize the initial bacterial adhesion [224–226]. These studies have shown that both calcium phosphate and carbonated hydroxyapatite loaded with antibiotics and used as coatings effectively inhibited the growth of* Staphylococcus aureus* [224–226]. Moreover, in an in vitro study, Liu et al. integrated simvastatin and metronidazole into a calcium phosphate coating for titanium surface and found that this bifunctional coating prevented the growth of the* Porphyromonas gingivalis* [227].

In addition to these coatings, biodegradable polymers and so-gel films have been proposed as controlled-release antibiotic-laden coatings on titanium surfaces. Specifically, Gollwitzer et al. prepared a biodegradable poly(D,L-lactic acid) coating with integrated gentamicin and teicoplanin on titanium surfaces and found a statistically significant reduction in the bacterial adhesion of* Staphylococcus epidermidis* when compared to the uncoated titanium alloys [228]. In other studies biodegradable poly(lactic-co-glycolic acid) coatings containing antibiotics were prepared by an electrospinning technique. The electrospinning process is based on the nanotechnology and produces polymeric nanofibers which can be used as drug delivery agents. These studies demonstrated that the antibiotic-loaded electrospun coating on titanium implants significantly reduced the adhesion of* Staphylococcus aureus* compared with the bare titanium implants in vitro and in vivo [229, 230]. However, the increase of microbial organisms resistant to antibiotics remains a considerable issue in the application of the drugs clinically. Moreover, some drug-containing coatings continue to release antibiotics at low concentrations for longer periods of time increasing the risk of antibiotic resistance. Finally, there are in vitro studies recording that some antibiotics provoke cell toxicity. Specifically, Ince et al. mentioned that the decreased osteoblastic activity may be attributed to the inhibition of protein synthesis provoked by the gentamycin at concentration greater than 100 *μ*g/mL [231]. Furthermore, Antoci et al. demonstrated a reduction in the osteoblast proliferation after the cells exposure to ciprofloxacin, tobramycin, and vancomycin even at the lowest dose of 25 *μ*g/mL for the ciprofloxacin. However, further in vitro and in vivo studies are required to assess the exact effect of the antibiotics on the human cells [231–233].

With the increase of microbial organisms resistant to several antibiotics, the application of nonantibiotic organic antimicrobial agents, such as chlorhexidine and chloroxylenol, has been investigated. Titanium surface has the capacity to absorb chlorhexidine and release it gradually over a long period of time [234, 235]. However, several studies have shown that the nonantibiotic organic antimicrobial agents can adversely affect human osteoblasts. Specifically, it was speculated that the chlorhexidine provoked lysis of the fibroblasts membrane leading to the cellular death. However, further investigation should be conducted in order for definitive conclusions to be drawn regarding the biocompatibility of the nonantibiotic antimicrobial agents [231, 236].

An alternative approach to the prevention of the bacteria adhesion is the modification of implants' surface characteristics. In vitro studies have shown that ultraviolet light irradiation (UV) treatment of Ti-6Al-4V prevents bacteria colonization [237, 238]. Moreover, some bioactive polymers, such as chitosan and hyaluronic acid, bonded to titanium demonstrated improved osteoblast attachment and inhibition of bacterial attachment [239, 240]. However, the in vivo performance of these molecules is not known.

#### 3.3.2. Inorganic Antimicrobial Coatings

One of the most promising and attractive approaches of obtaining antibacterial coatings for titanium is the incorporation of inorganic metallic antimicrobial agents on the titanium oxide layer [241–244]. Silver is a white, brilliant, and ductile metallic element with atomic number 47 in the periodic table. Pure silver has the highest electrical and thermal conductivity of all metals and possesses the lowest contact resistance [245]. The antimicrobial effect of silver (Ag) has been recognized since antiquity [246]. Other medical applications of Ag as coating for prevention of biofilm formation include the incorporation into bandages for cutaneous wounds, vascular, urinary and peritoneal catheters, prosthetic heart valve rings, vascular grafts, and sutures [247, 248].

Nanotechnology includes fields of science and technology and is based on the development of materials with dimensions in nanoscale level. Nanotechnology has a large range of applications such as in medicine, electronics, and biomaterials energy production. Nanoparticles are atoms between 1 and 100 nanometers in size, with enhanced chemical and mechanical properties. Among different types of nanomaterials, silver nanoparticles are considered to be the most antimicrobial against bacteria and viruses, due to their large surface area to volume ratio [249].

Regarding the antibacterial mechanism of Ag, the exact interaction between silver nanoparticles and bacteria is not known and thus there are many references proposing several possible mechanisms. It is believed that Ag binds to bacterial deoxyribonucleic acid (DNA), dissociating the hydrogen bonds between purine and pyrimidine bases and hence preventing the replication of DNA and cell division [250]. Another possible mechanism is the binding of silver to bacterial cell proteins and enzymes, such as the sulfhydryl groups leading to denaturation, disruption of the cell metabolism, and finally death [251, 252]. It has been reported that the extracellular binding of positively charged Ag nanoparticles to negatively charged peptidoglycans on bacteria walls is essential for the antimicrobial activity of Ag causing structural changes and cell atrophy [250]. Lastly, Slawson et al. believe that the antimicrobial mechanism of silver lays in the release of Ag ions into the cell, achieved by a transport system for molecules of similar charge and size [252].

Previous studies suggest that Ag nanoparticles, incorporated as a coating to titanium surfaces, are effective growth inhibitors of* Staphylococcus aureus* and* Escherichia coli*, preventing their adhesion or proliferation to these surfaces [243, 244]. Moreover, Jin et al. [253] investigated the antibacterial effect of zinc (Zn) and silver (Ag) coimplantation into titanium plates against* S. aureus* and* E. coli* both in vitro and in vivo and found a reduction in the bacterial growth on the Zn/Ag coimplanted titanium [253]. Another study examined the antibacterial activity of a biomimetic coating enriched by calcium (Ca), phosphorous (P), silicon (Si), and silver (Ag) nanoparticles and incorporated into the titanium surfaces. This study demonstrated a significant reduction in survivability of* Streptococcus mutans, Streptococcus epidermidis*, and* Escherichia coli* [254]. It should be mentioned however that the above examined strains are not representative of oral bacteria found in peri-implant disease [255]. Although there is no evidence for the presence of a limited number of specific bacteria in the peri-implantitis, in general, the microbiota associated with peri-implant disease is similar to the subgingival flora of chronic periodontitis and consists of* Porphyromonas gingivalis, Prevotella intermedia, Tannerella forsythia, Fusobacterium* sp.,* Actinomycetes, S. mutans, V. parvula, S. sanguinis*, and* S. gordonii* [255]. Nevertheless, the antimicrobial activity of Ag nanoparticles incorporated to titanium surfaces against these peri-implant bacteria has not been investigated thoroughly in the literature. Massa et al. examined the antibacterial effect of a silica-based composite coating containing Ag nanoparticles on the titanium surface against the* Aggregatibacter actinomycetemcomitans*, although Mombelli and Décaillet found that this microorganism is less frequently in the peri-implant diseases than the aforementioned bacteria [255, 256].

Besides the silver, other inorganic antimicrobial agents such as zinc (Zn), copper (Cu), and fluorine (F) have also been used as antibacterial coatings to titanium implants. However, silver is the most preferable, due to its broad antibacterial spectrum to both Gram-positive and Gram-negative bacteria, its long-lasting antibacterial effect, its biocompatibility, and its stability [257]. Previous studies have examined the antimicrobial effect of zinc [258, 259]. However, the reduction of the bacteria growth achieved by the zinc was not long-lasting [260]. Li et al. prepared coatings containing titanium (Ti) nanotubes and zinc (Zn) on titanium foils and found prevention on the bacterial colonization for 2 weeks [261]. However, it was found that this antibacterial action decreased with time. Moreover, there is evidence that the incorporation of copper (Cu) into the titanium alloys increases their antimicrobial effect. It should be mentioned however that copper ion implantation compromises the mechanical properties of the metals reducing their corrosion and wear resistance [262, 263].

## 4. Conclusion

Titanium and its alloys are a very promising biomaterial for the fabrication of medical and dental implants, due to the excellent mechanical properties and biocompatibility. The introduction of *β*-phase stabilizers and bulk metallic glasses in the Ti-alloys has enabled the development of new alloys with high mechanical strength and low modulus of elasticity that allows proper loading of the bone. Furthermore, the high corrosion resistance and biocompatibility of these new alloys will limit failures such as fatigue fractures, implant loosening, and adverse reactions due to ion release. Additionally, porous surfaces and bioactive implant coatings seem to improve the bioactivity of implants and lead to faster and more enhanced osseointegration. Moreover, surface coatings with antimicrobial effects are very promising for limiting failures due to infection.

Although much progress has been made it seems that there are still many improvements to be achieved. Fabrication methods and their parameters appear to play a pivotal role on the mechanical properties, corrosion resistance, pore size, and distribution of the materials. Furthermore, cellular interactions with the modified implant surfaces have to be fully understood at a nanometer level, in order to manufacture implants with high osseointegration rates and strong antibacterial effects. The findings of the current literature are very promising, but there is still room for improvement for implants that will provide a better quality of life to both medical and dental patients.

## Figures and Tables

**Table 1:** 

Porosity	64%	76%

Young's modulus	3.3 ± 0.8 GPa	2.1 ± 0.5 GPa

Compressive strength	102 ± 10 MPa	23 ± 10 MPa

**Table 2:** 

*β*-alloys content	Elastic modulus (GPa)	Elastic modulus after aging (GPa)
Ti-15Zr [102]	112	NR
Ti-15Zr-4Nb-4Ta-0.2Pd-0.2O-0.05N [151]	100	97
Ti-10Zr [102]	95	NR
Ti-15Zr-4Nb-4Ta-0.2Pd [151]	94	99
Ti-16Nb-13Ta-4Mo [97]	91	113
Ti-15Sn-4Nb-2Ta-0.2Pd [151]	89	103
Ti-5Zr [102]	87	NR
Ti-15Sn-4Nb-2Ta-0.2Pd-0.2O [151]	86	98
Ti-15Zr-10Cr [99]	80	NR
Ti-29Nb-13Ta [97]	76	103
Ti-15Mo [102]	75	NR
Ti-13Nb-13Zr [102]	75	NR
Ti-29Nb-13Ta-4Mo [97]	74	73
Ti-29Nb-13Ta-6Sn [97]	74	73
Ti-29Nb-13Ta-2Sn [97]	62	78
Ti-19Zr-10Nb-1Fe [98]	59	NR
Ti-29Nb-13Ta-7Zr [102]	53	NR
Ti-10Zr-5Nb-5Ta (ARB processed) [104]	43	NR
